# Proteomics Landscape of Host-Pathogen Interaction in *Acinetobacter baumannii* Infected Mouse Lung

**DOI:** 10.3389/fgene.2021.563516

**Published:** 2021-05-07

**Authors:** Xin Li, Xiaofen Liu, Peter Horvatovich, Yingwei Hu, Jing Zhang

**Affiliations:** ^1^Institute of Antibiotics, Huashan Hospital, Fudan University, Shanghai, China; ^2^Key Laboratory of Clinical Pharmacology of Antibiotics, National Health and Family Planning Commission, Shanghai, China; ^3^National Clinical Research Center for Aging and Medicine, Huashan Hospital, Fudan University, Shanghai, China; ^4^Department of Analytical Biochemistry, Groningen Research Institute of Pharmacy, University of Groningen, Groningen, Netherlands; ^5^Department of Pathology, Johns Hopkins University, Baltimore, MD, United States

**Keywords:** *Acinetobacter baumannii*, lung infection, host response, NADPH oxidase, TMT-labeling proteomics

## Abstract

*Acinetobacter baumannii* is an important pathogen of nosocomial infection worldwide, which can primarily cause pneumonia, bloodstream infection, and urinary tract infection. The increasing drug resistance rate of *A. baumannii* and the slow development of new antibacterial drugs brought great challenges for clinical treatment. Host immunity is crucial to the defense of *A. baumannii* infection, and understanding the mechanisms of immune response can facilitate the development of new therapeutic strategies. To characterize the system-level changes of host proteome in immune response, we used tandem mass tag (TMT) labeling quantitative proteomics to compare the proteome changes of lungs from *A. baumannii* infected mice with control mice 6 h after infection. A total of 6,218 proteins were identified in which 6,172 could be quantified. With threshold *p* < 0.05 and relative expression fold change > 1.2 or < 0.83, we found 120 differentially expressed proteins. Bioinformatics analysis showed that differentially expressed proteins after infection were associated with receptor recognition, NADPH oxidase (NOX) activation and antimicrobial peptides. These differentially expressed proteins were involved in the pathways including leukocyte transendothelial migration, phagocyte, neutrophil degranulation, and antimicrobial peptides. In conclusion, our study showed proteome changes in mouse lung tissue due to *A. baumannii* infection and suggested the important roles of NOX, neutrophils, and antimicrobial peptides in host response. Our results provide a potential list of protein candidates for the further study of host-bacteria interaction in *A. baumannii* infection. Data are available via ProteomeXchange with identifier PXD020640.

## Introduction

*Acinetobacter baumannii* is a hazardous Gram-negative opportunistic pathogen of nosocomial infection that represent serious health risk worldwide. The most serious consequence of lung infection by *A. baumannii* is nosocomial pneumonia, which has high mortality rate. In recent years, the resistance of *A. baumannii* to antibiotics is prominently increasing (Hu et al., 2019), and the resistance rate against imipenem and meropenem in China were 73.6 and 75.1% in 2019, respectively^1^. Although colistin is used as a last-line antibiotic against multi-resistant *A. baumannii*, the nephrotoxicity of this compound greatly limited its clinical use (Li et al., 2006). The prevalence of resistant *A. baumannii* and the slow process of the discovery of new antibiotics has brought great challenges in clinical treatment and highlight the importance of developing new effective strategies for *A. baumannii* infections.

The innate immune system plays a critical role in the host defense against *A. baumannii* (Garcia-Patino et al., 2017). A better understanding of immune response to *A. baumannii* infection can facilitate the development of immune-adjunctive therapies. Some studies about the pathogenesis and host immune defense mechanisms against *A. baumannii* have been reported (Bruhn et al., 2015). Endothelial cells are the first line of defense against bacterial challenges (Feng et al., 2014). TLR4, mainly located on the surface of myeloid original cells such as monocytes, macrophages, microglia, myeloid DCs, and granulocytes, is the main receptor, which recognizes lipopolysaccharides (LPS) of Gram-negative bacteria, activating NF-κB signaling pathway, leading to the production of cytokines and chemokines (Kim et al., 2013; Kawasaki and Kawai, 2014; Noto et al., 2015). Neutrophils can be recruited to the infected site by cytokines, which then engulf the pathogens recognized by the surface receptors, finally kill them through the production of reactive oxygen species (ROS) and secretion of antimicrobial peptides (van Faassen et al., 2007). Some other molecular/signaling mechanisms of infection response were also under investigation, such as neutrophil extracellular traps (NETs) (Kamoshida et al., 2015), IL-17 signaling pathway (Breslow et al., 2011), and inflammasome NLRP3 (Dikshit et al., 2018). However, most of these studies focused on specific proteins or pathways, ignoring the complicated networks of the human immune response. Hence, analyzing the global level responses will advance our understanding of host-pathogen interactions.

The rapid development of high-throughput mass spectrometry provides an effective analytical quantitative profiling platform for the systematic study of proteome changes of host response. Ferrer-Navarro et al. compared the proteome profiles of mouse lungs infected by *Streptococcus pneumoniae* (Gram-positive bacteria) with control mice at 24 and 48 h post-infection by 2D-differential gel electrophoresis (2D-DIGE). 91 differentially expressed proteins were identified, and the analysis showed that the cytoskeleton of host lung tissue cells is modified in *S. pneumoniae* infection (Ferrer-Navarro et al., 2018). Seddigh et al. (2017) applied a label-free proteome analysis of alveolar epithelial cells infected with *A. fumigatus* (fungus), which study revealed the key role of IL-41 in the host defense. Varnum et al. (2012) showed that the expression levels of numerous host proteins in bronchoalveolar lavage fluid (BALF) are altered in response to mice pulmonary infection with *Francisella tularensis novicida* (Gram-negative bacteria), and specific early infection biomarkers were identified. Currently, the molecular mechanisms of host-pathogen interaction in the *A. baumannii* infected lung tissue are poorly understood and need detailed investigations. Tandem mass tag (TMT) proteomics is a widely used proteomics approach that allows high sample multiplexing and provides more accurate quantification of protein changes between samples than label-free approach by excluding some of the technical variability related to sample preparation. In the present work, 10-plex TMT-labeling quantitative proteomic by LC-MS/MS was employed to identify the differentially expressed proteins in mice lung tissue infected by *A. baumannii*, and bioinformatic analysis was conducted to provide proteomic evidence for the host immune process.

## Materials and Methods

### Bacterial Strain and Animals

The *A. baumannii* strain ATCC 19606 was purchased from the American Type Culture Collection. Bacteria was cultured on a Mueller-Hinton agar (Oxoid, United Kingdom) plate and grown for 18–22 h at 35°C. A single colony was selected and subcultured in Muller-Hinton broth and grown to OD_600 *nm*_ 0.2.

Specific-pathogen-free (SPF) female ICR mice (Sipper-BK, Shanghai, China), 24–26 g, were used in the lung infection model. The experiments were approved by the Experimental Animal Ethics Committee of Pharmacy, Fudan University (2019-03-HSYY-ZJ-01) and followed the Experimental Animal Welfare Review Guide.

### Lung Infection Model

30 mice were randomly separated into two groups: control group (CON, *n* = 9) and infection group (INF, *n* = 21), and were anesthetized with an intraperitoneal injection of 25 mg/kg 2,2,2-tribromoethanol. The mice in INF group were intranasally inoculated with 50 μL of *A. baumannii* suspension (10^7^ CFU/mL), and the CON group received 50 μL saline solution.

For the proteomic study, both CON and INF groups had three replicates, each replicate mixed lung tissues from three different mice. Hence, 18 mice from both animal groups were sacrificed at 6 h after inoculation and lungs were collected and washed twice in saline and stored at −80°C before protein extraction.

For the bacteria counting in lungs in the INF group, 12 mice were sacrificed at 2, 6, 12, and 24 h after bacteria inoculation (three replicates for each time point). Specifically, lungs were removed aseptically and homogenized in 1.8 mL sterile saline using homogenizer (France, Bertin Technologies, Bertin Precellys 24). The homogenate was serially diluted with sterile saline and 100 μL of diluent was cultured on Muller-Hinton agar at 37°C for 18–22 h. The number of CFU was counted as Log_10_ (CFU/lung) in lung tissue.

### Extraction and Trypsin Digestion of Lung Proteins

The lung tissues were ground into cell powder in liquid nitrogen and four volumes of lysis buffer (8 M urea, 1% protease inhibitor cocktail) were added to the powder, followed by sonication three times on ice using a high-intensity ultrasonic processor (Scientz). The remaining debris was removed by centrifugation at 12,000 g at 4°C for 10 min. The supernatant was then collected and the protein concentration was determined with BCA kit (Beyotime) according to the manufacturer’s instructions. For digestion, the protein solution was reduced with 5 mM dithiothreitol for 30 min at 56°C and alkylated with 11 mM iodoacetamide for 15 min at room temperature in darkness. The protein sample was then diluted by adding 100 mM triethylamonium bicarbonate (TEAB) until the urea concentration reached 2 M. Finally, trypsin was added at 1:50 trypsin-to-protein mass ratio for the first digestion overnight and 1:100 trypsin-to-protein mass ratio for a second 4 h digestion.

### 10-Plex TMT Labeling

After trypsin digestion, the peptide was desalted using Strata X C18 SPE column (Phenomenex) and vacuum-dried. Peptides were reconstituted in 0.5 M TEAB and labeled according to the manufacturer’s protocol for TMT kit (Thermo). Briefly, one unit of TMT reagent was thawed and reconstituted in acetonitrile, then mixed with the peptide digest and incubated for 2 h at room temperature. All the labeled peptides were pooled, desalted and dried during centrifugation in vacuum before LC-MS/MS analysis.

### LC-MS/MS Analysis

The mixed 10-plex TMT-labeled tryptic peptides were fractionated using reverse-phase HPLC with an Agilent 300 Extend C18 column (5 μm, 4.6 mm ID, 250 mm length). The peptides were separated with a gradient of 8–32% acetonitrile (pH 9.0) over 60 min into 60 fractions and combined into 18 fractions and dried by vacuum centrifugation. The fractionated peptides were redissolved in 0.1% formic acid (solvent A) and injected into Q Exactive^TM^ Plus (Thermo) coupled online to an EASY-nLC 1000 UPLC platform for the proteomic analysis. The peptides were separated by a home-made reversed-phase analytical column (15 cm length, 75 μm i.d.). The gradient was started with an increase of solvent B from 5 to 22% (0.1% formic acid in 98% acetonitrile) over 40 min, continued increasing from 22 to 35% in 13 min, and finally increased to 80% in 3 min and held for 4 min. The flow rate was constant set as 800 nL/min and the solvent A consisted of 0.1% formic acid aqueous solution.

A data-dependent acquisition (DDA) consisted of duty cycle starting with one single-stage MS scan and followed by 20 MS/MS scans with 15.0 s of dynamic exclusion time of a fragmented precursor m/z. Automatic gain control (AGC) was set at 5 × 10^4^. MS1 spectra were collected in the range of 350–1,800 m/z at a resolution of 70,000, and MS2 spectra were collected at a resolution of 17,500 and the fixed first mass was set as 100 m/z. The isolation width for MS2 scan was 2.0 Da. The collision energy was set as 28%.

### Database Search and Statistical Analysis

The MS/MS spectra data were processed using MaxQuant (v.1.5.2.8). Tandem mass spectra were searched against the SwissProt Mouse database (29,795 sequences) concatenated with reverse decoy database. Trypsin/P was specified as cleavage enzyme allowing up to 2 missed cleavages. The mass tolerance for precursor ions was set as 20 ppm in the first search and 5 ppm in the main search, and the mass tolerance for fragment ions was set as 0.02 Da. Carbamidomethyl on Cys was set as fixed modification and oxidation on Met residue was specified as variable modifications. FDR was adjusted to < 1% at PSM, peptide and protein levels and the minimum score for peptides was set > 40. The correction for isotope cross contamination has been performed in MaxQuant based on data provided by Thermo Scientific.

Statistical analysis was performed with the *t*-test using R software version 3.5.1. Given that the fold change compression of TMT has made the observed differences smaller compared to the real differences, a cutoff of the nominal *p* < 0.05 was considered to be significant and the fold change > 1.2 or < 0.83 was required to identify proteins differentially expressed.

### Bioinformatics Analysis

The STRING Database^2^ and gProfiler^3^ were used to analyze the differentially expressed proteins for Gene Ontology (GO) annotation, KEGG and Reactome pathway analysis. A dataset of all the differentially expressed protein identifiers was uploaded to STRING and *Mus musculus* was chosen as organism classification. The minimum required interaction score was 0.4 for each protein-protein association.

### Validation

Lung of six mice were used for Elisa validation. Three mice in control group and the other three in infection group. The MMP-9 Elisa kit (R&D, MMPT90, the detection range was 0.08∼5 pg/mL) and S100a8/a9 Elisa kit (R&D, DY8596-05, the detection range was 125∼5,000 pg/mL) were used according to the manufacturer’s instructions to determine the concentration of proteins in the tissue homogenate. The proinflammatory factor IL-6 (R&D, DY406-05, the detection range was 15.6∼1,000 pg/mL) was also determined to show the host inflammatory response.

## Results

### Bacteria Counting After Infection

**FIGURE 1:**
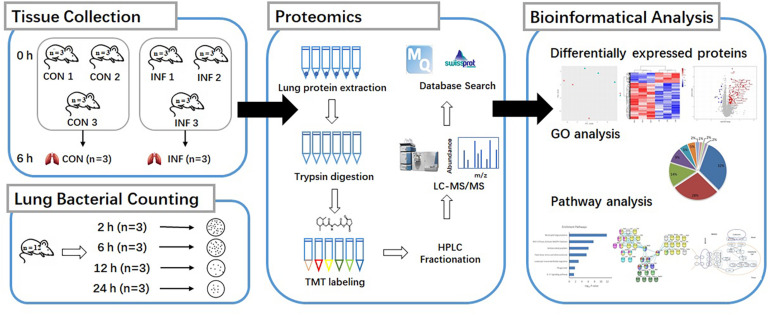
Experimental setup, sample preparation and bioinformatic workflow for comparison proteome of *A. baumannii* infected mice lung tissue (INF) with the control group (CON).

**FIGURE 2:**
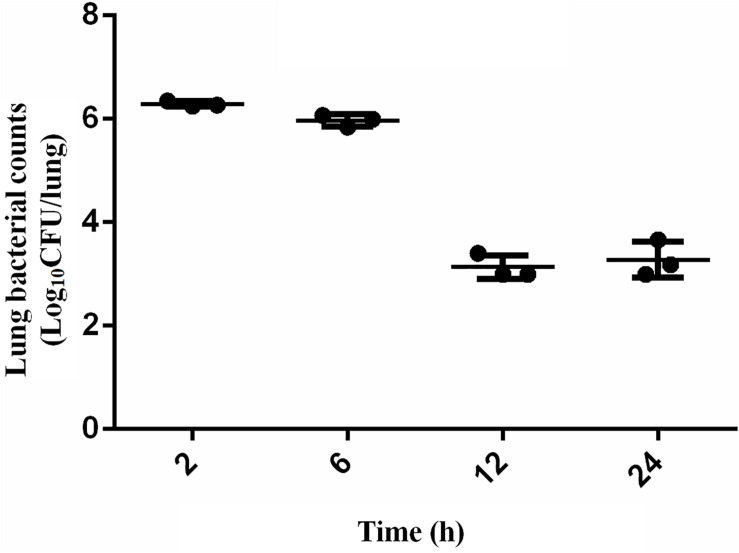
Bacteria counting in the lungs of mice after intranasally infected with 5 × 10^5^ CFU *A. baumannii* within 24 h. Data presented as mean ± *SD*. In murine lung infection model, a 2-log_10_ CFU kill could be considered a good bactericidal effect. A 3.2 log_10_CFU bacterial reduction was observed in lungs 12 h after infection compared with that at 2 h in this study, which could be considered as a significant and effective decrease.

The scheme of the experimental design is shown in Figure 1. Mice were randomly separated into two categories: the proteomic and bacteria counting categories. 18 mice were designated into proteomic category and separated into control group (CON, *n* = 9) and infection group (INF, *n* = 9). Lung tissues were collected from each mice and pooled to prepare three replicate samples, using three animals per pool. The rest of mice (*n* = 12) were in the bacteria counting category, in which they were infected with bacteria and sacrificed at 2, 6, 12, and 24 h after bacteria inoculation (three replicates for each time point). Bacterial load at the lung, 2 h post-infection, was 6.29 ± 0.05 log_10_ CFU/lung tissue after intranasally infected with 1 × 10^5^ CFU/lung (5.7 log_10_CFU/lung) *A. baumannii*. It remained basically unchanged at 6 h (5.97 ± 0.12 log_10_ CFU/lung). However, the bacterial counts at 12–24 h decreased to 3.13 ± 0.23 and 3.28 ± 0.34 log_10_ CFU/lung, respectively (Figure 2).

### Proteomic Expression Patterns in *A. baumannii*-Infected Mice

**FIGURE 3:**
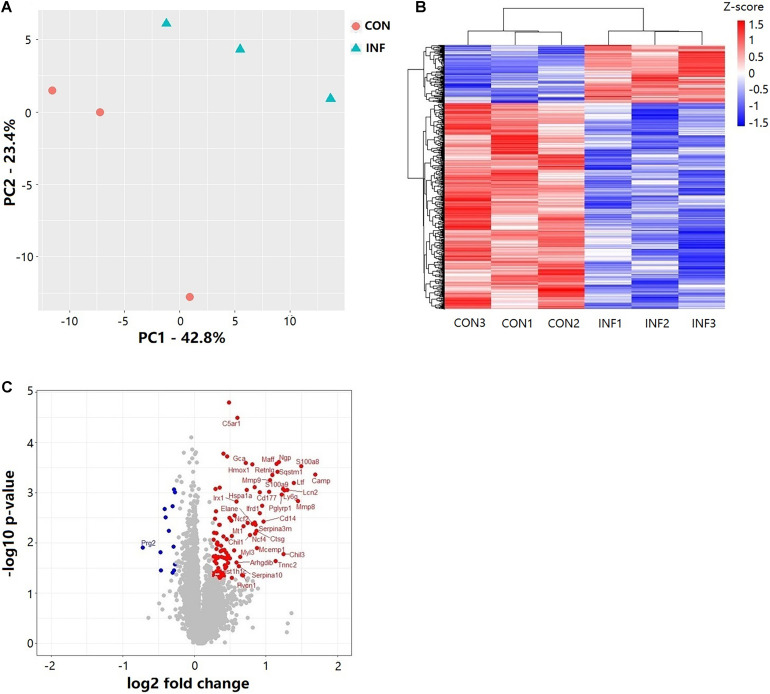
Results of TMT label quantitative proteome analysis of mice lungs. **(A)** PCA score plots of the first two principal components of proteomic expression levels in mice lungs of CON and INF groups. Each group has 3 biological replicates, each replicate composes of three different mice. **(B)** Hierarchical clustering of proteins with Student’s *t*-test with nominal *p* ≤ 0.05. **(C)** Volcano plot showing the differential proteins up and down regulated in INF group. Student’s *t*-test nominal *p* ≤ 0.05 and a ratio of means ≥ 1.2 or ≤ 0.83 are applied as significance thresholds. Differentially expressed proteins are highlighted in red (up-regulated) or blue (down-regulated). The gene names are given for the proteins whose ratio ≥ 1.5 or ≤ 0.67.

Overall, 6,218 proteins were identified in the two groups (CON and INF) in which 6,172 could be quantified with reporter ion intensity detected in at least one MS/MS spectra. As shown in the principal component analysis (PCA) score plots and heatmap, the *A. baumannii* infection induced significant proteome changes 6 h after inoculation (Figures 3A,B). Proteins with relative expression level fold change > 1.2 or < 0.83 were considered as differentially expressed proteins (DEPs, *p* < 0.05). The volcano plots for the DEPs are shown in Figure 3C. There were 121 DEPs between the INF and CON mice groups, with 108 up-regulated and 13 down-regulated proteins in the INF group. The full list of differentially expressed proteins identified between CON and INF sample groups of 18 mice lung can be found in Supplementary Table 1.

### Functional Characterization of Differentially Expressed Proteins

**FIGURE 4:**
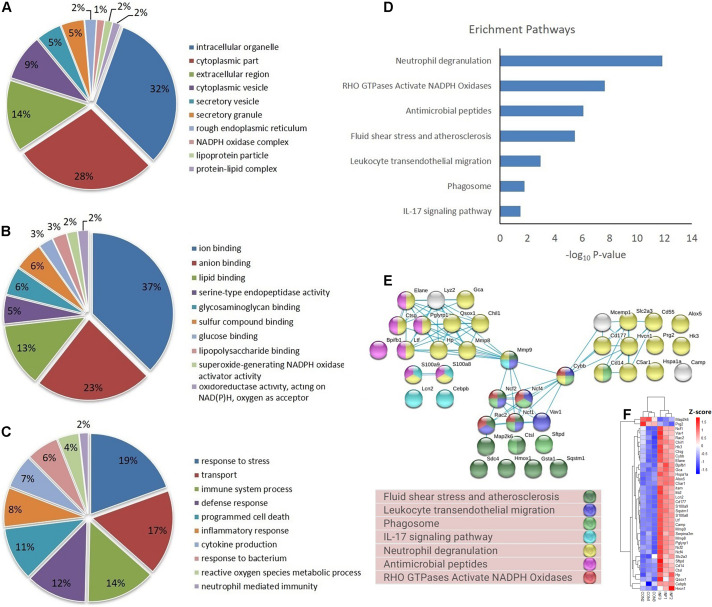
GO, enrichment pathways and protein-protein interactions of DEPs. **(A)** Cellular component (GO analysis); **(B)** molecular function (GO analysis); **(C)** biological process (GO analysis); **(D)** pathways (KEGG and REACTOME); **(E)** protein-protein interaction using STRING database; **(F)** expression of proteins involved in key pathways.

Based on GO database, the DEPs were classified into different biological process, cellular component and molecular function (Figures 4A–C). The DEPs were mainly located in cytoplasm, intracellular organelle, membrane, and extracellular region, and they were mainly involved in the molecular function of ion binding, lipid binding and serine-type endopeptidase activity. For biological process, the DEPs were involved in response to stress, transport, immune system process and programmed cell death. The most important GO categories included cytokine production (GO.0001816, CD14, Camp, Mmp8, Pglyrp1, Elane), antimicrobial humoral defense (GO.0019730, Camp, Ltf, Sftpd, Ctsg), NADPH oxidase complex (GO.0043020, Cybb, Ncf1, Ncf2, Ncf4), and apoptotic process (GO.0006915, S100a8, S100a9, Lcn2, Hmox1).

KEGG pathway analysis of DEPs revealed that multiple immune-related pathways are involved in the host responses including fluid shear stress and atherosclerosis, leukocyte transendothelial migration, phagocytosis, and IL-17 signaling pathways. A search using the Reactome database revealed DEPs participating in signaling pathways such as neutrophil degranulation, RHO GTPases Activate NADPH oxidases and antimicrobial peptides (Figure 4D). STRING protein-protein interaction database was used to further explore the interactions between proteins in significantly enriched pathways. STRING analysis showed that the proteins were divided into three main protein-protein interaction clusters. Figure 4E shows that the tight protein-protein clusters reflected consistently enriched pathways, except for neutrophil degranulation, which was present in multiple clusters, which is possibly due to diverse functions of neutrophil secreted proteins. Multiple proteins participated in more than one pathways, especially Mmp9, Cybb, Ncf1, Ncf2, Ncf4, and Rac2. It’s worth mention that both Cybb and Mmp9 participate in four pathway and link together multiple clusters, which suggested their important role in the lung response to *A. baumannii* infection. The 38 DEPs (Table 1) part of key pathways are shown in Figures 4D–F including neutrophil degranulation, RHO GTPases Activate NADPH oxidases and antimicrobial peptides. The details of the GO, KEGG and REACTOME pathways of all the differentially expressed proteins between INF and CON groups were shown in Supplementary Table 2.

**TABLE 1:** DEPs involved in key pathways.

Uniprot ID	Gene name	Protein name	Expression fold change	−log_10_ *p*-value
P30993	C5ar1	Complement component 5a receptor 1	1.52	4.49
P48999	Alox5	Arachidonate 5-lipoxygenase	1.38	3.72
Q8VC88	Gca	Grancalcin	1.65	3.59
P27005	S100a8	S100 calcium binding protein A8 (calgranulin A)	2.82	3.53
Q64337	Sqstm1	Sequestosome 1	2.23	3.41
P51437	Camp	Cathelicidin antimicrobial peptide	3.23	3.36
P41245	Mmp9	Matrix metallopeptidase 9	2.08	3.25
P08071	Ltf	Lactotransferrin	2.62	3.19
P31725	S100a9	S100 calcium binding protein A9 (calgranulin B)	2.36	3.07
Q61696	Hspa1a	Heat shock protein 1A	1.66	3.06
P11672	Lcn2	Lipocalin 2	2.46	3.05
Q8R2S8	Cd177	CD177 antigen	2.07	3.02
Q61114	Bpifb1	BPI fold containing family B, member 1	6.28	2.96
O88593	Pglyrp1	Peptidoglycan recognition protein 1	2.34	2.96
O70138	Mmp8	Matrix metallopeptidase 8	2.73	2.83
P05555	Itgam	Integrin subunit alpha M	1.93	2.74
P11835	Itgb2	Integrin subunit beta 2	1.47	2.54
Q61093	Cybb	Cytochrome b-245, beta polypeptide	1.43	2.44
P10810	Cd14	CD14 antigen	1.95	2.43
O70145	Ncf2	Neutrophil cytosolic factor 2	1.67	2.40
Q3UP87	Elane	Elastase, neutrophil expressed	1.76	2.38
Q03734	Serpina3m	Serine (or cysteine) peptidase inhibitor, clade A, member 3M	1.81	2.36
Q61646	Hp	Haptoglobin	1.27	2.36
P28293	Ctsg	Cathepsin G	1.83	2.24
P06797	Ctsl	Cathepsin L	1.21	2.20
P97369	Ncf4	Neutrophil cytosolic factor 4	1.80	2.19
Q61362	Chil1	Chitinase-like 1	1.71	2.16
Q3TRM8	Hk3	Hexokinase 3	1.43	2.13
P70236	Map2k6	Mitogen-activated protein kinase kinase 6	0.82	1.93
Q61878	Prg2	Proteoglycan 2, bone marrow	0.61	1.91
Q09014	Ncf1	Neutrophil cytosolic factor 1	1.30	1.72
Q05144	Rac2	Rac family small GTPase 2	1.41	1.69
P27870	Vav1	Vav 1 oncogene	1.22	1.66
P50404	Sftpd	Surfactant associated protein D	1.35	1.56
P32037	Slc2a3	Solute carrier family 2 (facilitated glucose transporter), member 3	1.36	1.53
Q8BND5	Qsox1	Quiescin Q6 sulfhydryl oxidase 1	1.29	1.42
Q3U2S8	Hvcn1	Hydrogen voltage-gated channel 1	1.60	1.35
P28033	Cebpb	CCAAT/enhancer binding protein (C/EBP), beta	1.26	1.32

### Validation

**FIGURE 5:**
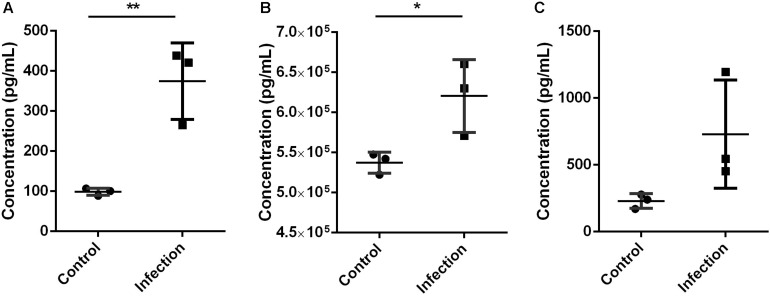
Expression level of Mmp9 **(A)**, S100a8/a9 **(B)**, and IL-6 **(C)** in mice lung tissue 6 h after inoculated with *A. baumannii* by Elisa. MMP9 and S100a8/a9 showed the same expression pattern with proteomics analysis. Values represent the means ± SD of three replicates. Shapiro-Wilk test was performed on the Elisa data that showed the data followed a normal distribution (*p* > 0.05), so *t*-test was employed to compare the Elisa data between the two groups. Student’s *t*-test, *p < 0.05; **p < 0.01.

The validation using Elisa kits found that key proteins Mmp9 and S100a8/a9 were significantly increased in mouse lung tissue during *A. baumannii* clearance (Infection versus Control, nominal *p* < 0.05, Figures 5A,B). This result is in agreement with the protein levels measured by TMT analysis. The expression level of IL-6 was higher at 6 h after infection than that in control mice, although with no significant difference (Infection versus Control, nominal *p* = 0.10, Figure 5C). It also suggested the occurrence of immune response.

## Discussion

*A. baumannii* is one of the major pathogens endangering public health where the host immune system plays as an important barrier, and the infection usually occurs when the host is immunocompromised. In the treatment of *A. baumannii* infection, the development of drug resistance leads to an important lack of clinically selectable drugs, and immune-related treatment regimens enforcing host immune response are taken into consideration. The mechanism of the host response to *A. baumannii* has not been elucidated. In this study, we firstly described the proteome changes of mice lung tissue during the clearance of *A. baumannii*, and analyzed their functions as well as involved pathways.

Bacterial counts in the lung tissue of mice showed a decrease of bacteria between 6 and 12 h after infection, and the up-regulation of a large number of immune-related proteins demonstrated the immune response. The increased level of IL-6 also suggested the immune response, although with no significant difference possibly due to the inappropriate sampling time. We observed a significant up-regulation of proteins involved in receptor recognition (CD14, S100a8, S100a9, Itgb2, Itgam). CD14 is a GPI-anchored cell surface molecule that functions as a receptor for LPS, the major cell membrane component of Gram-negative bacteria, and upon contact with the bacterial surface transfers the subsequent molecular response to eliminate the pathogens. The binding of LPS to CD14 plays a key role in innate immune against *A. baumannii* via LPS moiety which helped to effectively eliminate the bacteria from lungs (Knapp et al., 2006; Park and Lee, 2013). S100a8 and S100a9 are subunits of an antimicrobial heterodimer, calprotectin. Imaging MS showed a strong correlation between calprotectin expression and bacterial burden in *A. baumannii* pneumonia mice, which can be used as a marker of inflammatory response (Moore et al., 2014). S100A9^–/–^ mice had higher mortality and bacterial burden compared with the wildtype after infected with *A. baumannii*. The reason of this higher mortality can be because calprotectin could inhibit *A. baumannii* growth through the chelation of Mn and Zn. This calprotectin-meditated Zn sequestration also participates in the *A. baumannii* drug resistance development through the influence on Zn acquisition system (Hood et al., 2012). Besides, S100a9 was indicated that it can bind CD14 functioning as a co-receptor for the S100a9-mediated TLR4-signaling pathway (Haziot et al., 2016). Igtam/Itgb2, also known as CD11b/CD18, Mac-1 or CR-3, is a surface receptor integrin expressed on many innate immune cells including monocytes, granulocytes and macrophages (Hughes et al., 1992; Zhou et al., 2013). *A. baumannii* inhibits neutrophil-induced NET formation by suppressing the adhesion ability of neutrophils, partly due to suppression CD11a expression but not of the CD11b expression on neutrophil surface (Kamoshida et al., 2018). Our study similarly detected an increased expression of CD11b, indicating that CD11b is not associated with the decreased adhesion in NET formation.

Proteins involved in anti-microbial defense (Camp, Ltf, Ctsg, Sftpd) were also up-regulated during the bacteria clearance process. Cathelicidin antimicrobial peptide (Camp) serves as a component of innate response and they are mainly stored in neutrophil and macrophage granules. The amphophilic property of Camp enables its accurate adhesion and anchoring, embedding in the lipid bilayer and creating transmembrane pores (Kosciuczuk et al., 2012), which leads to bacteria-killing. Recently, it has been shown that a variety of antimicrobial peptides have good antibacterial activity against multi-drug resistant *A. baumannii* and can be used as a potential therapy (Barksdale et al., 2017; Liu et al., 2017; Spencer et al., 2018). Lactotransferrin (Ltf) widely occurs in the blood and biological fluid. Upon inflammatory stimulates, Ltf can be recruited and delivered at the inflammatory site by neutrophils and released in iron-free form so that its anti-microbial activity can be activated through iron scavenging property. Colistin-induced LPS-deficient *A. baumannii* presented with increased sensitivities to lysozyme and lactoferrin *in vitro* (Kamoshida et al., 2020), and the combination of lactoperoxidase and lactoferrin also showed antibacterial activity in mice with *A. baumannii* pneumonia (Mahdi et al., 2018). Cathepsin G (Ctsg) is a serine protease that is involved in direct intracellular killing process of phagocytosed bacteria with myeloperoxidase (MPO) and ROS. Besides, it participates in extracellular killing of bacteria by modifying DNA structure of the pathogen, which serves as a component of NETs. Neutrophil-derived Ctsg critically contributes to the deceleration of pathogen replication during the *mycobacterial* lung infection of mice (Steinwede et al., 2012), and Ctsg can be potently inhibited by *Staphylococcus aureus* immune evasion proteins (Herdendorf et al., 2020). There are few reported studies relevant to the Ctsg effect on *A. baumannii*. Surfactant protein D (Sftpd) is reported to be an important component of the immune system of the lung. It can recognize LPS of Gram-negative bacteria, leading to the formation of CRD (Carbohydrate recognition domain)-dependent bacterial aggregation which enhances the binding to its receptors such as CD14 (Crouch and Wright, 2001). This binding process provides a potential treatment of bacterial infection diseases (Reinhardt et al., 2016). We found these proteins up-regulated indicating their important roles in the clearance of *A. baumannii* from mouse lung tissue. Ten abundant proteins involved in Antimicrobial peptides pathway (REAC:R-MMU-6803157), suggesting that antimicrobial peptide is a key killing mechanisms of *A. baumannii.*

**FIGURE 6:**
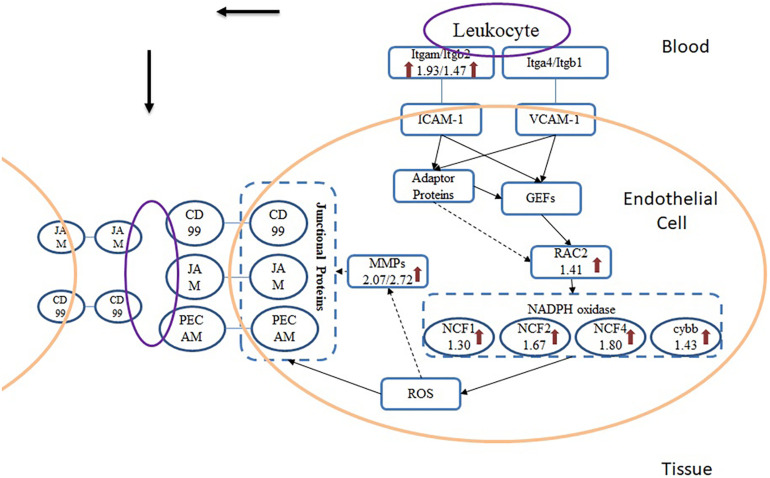
Scheme of TEM pathway during *A. baumannii* infection. Arrows indicate the proteins that were significantly up-regulated and the numbers beneath the gene names represent fold change.

From the results of pathway analysis, we found that Leukocyte Transendothelial Migration (TEM) and Phagosome pathway were activated. TEM refers to the process that leukocyte crosses blood vessel barrier from blood to the inflammatory site. After infected with *A. baumannii* strain ATCC19606, eight proteins in the lung of mice involved in TEM were significantly up-regulated (Figure 6), and they were related to multistep function of TEM pathway including recognition, adhesion, signal transduction in endothelial cells and transmigration. TEM is currently regarded as a multistep process including leukocyte capture, firm adhesion, crawling and transmigration in paracellular and transcellular routes, modulated by inflammatory cytokines and intracellular signaling events (Nourshargh et al., 2010; Vestweber, 2010; Muller, 2011; Schimmel et al., 2017). The initial adhesion of leukocyte and endothelial cell (EC) dependent on the interaction between P- or E-selectin on ECs and their ligands, which induces the cluster of EC receptors including ICAM-1 and VCAM-1 (Langer and Chavakis, 2009). In the TEM process, Itgam expressed on the surface of leukocyte serves as a ligand interacting with ICAM-1 on the endothelial cells. This binding induces the recruitment and phosphorylation of actin-binding adaptor proteins such as cortactin, leading to actin polymerization and recruitment of more ICAM-1 to the site of leukocyte adhesion (Nourshargh et al., 2010). The cluster of ICAM-1 protein on endothelial cell surface also induces the intracellular release of Ca^2+^ and activation of GEF (Guanine nucleotide exchange factors) which drives activation of Rho family proteins including RhoA, Rac and Rho GTPase. Rac serves as the activator for NADPH oxidase (NOX), producing ROS that can induce the reduction of cell-cell contact on ECs and support the direct killing of ingested pathogens. Rac protein subfamily includes Rac1, Rac2, and Rac3 in which Rac1 and Rac2 were mostly studied. Neutrophils from Rac2 deficient mice while express normal levels of Rac1 demonstrate deficits in multiple functions including superoxide production (Roberts et al., 1999). We found only Rac2 significantly up-regulated in *A. baumannii* infected mice lung without change in Rac1 level, which also indicates that Rac2 is the predominant Rac species involved in the innate immune response rather than Rac1, especially for *A. baumannii* infection. ROS is generated by the activation of NOX, which subsequently passes the signaling to endothelial cells that leads to the loosening of adhesion junctions and opening of transient channels across ECs for leukocyte transcellular migration. Junctional proteins including JAM, PECAM-1 and CD99 are activated by ROS during infection and bind the leukocyte ligands such as MAC-1, leading to vascular permeability (Muller, 2011). There were no significant changes of junctional proteins in lung tissue after *A. baumannii* infection, but MAC-1, the ligand for JAM, is increased in abundance. Besides, we found an obvious change of MMP-9 which is activated by ROS and have the function to cleave the endothelial cell junctions (Vermeer et al., 2009), leading to the weakening of cell-cell adhesion and opens a channel through epithelia which leukocyte can migrate.

Phagocytosis occurs in specialized cells such as macrophages, neutrophils and dendritic cells which functions as a critical process of bacteria-killing and clearance of cell debris. This process is initiated by the recognition and binding of the pathogen by the phagocyte surface, which triggers the cytoskeletal reorganization, leading to the uptake of pathogen and formation of phagosome (Aderem, 2003). After encapsulated in phagosomes, the cell kills pathogens using ROS, nitrogen oxide (NO) or antibacterial peptides released from the granules. Different from neutrophils, macrophages kill pathogens through the synthesis of NO with cytostatic and cytotoxic activity against bacteria (Dale et al., 2008). Drug resistance may affect bacterial susceptibility to phagocytosis. Sato et al. demonstrated renewed virulence characteristics in multi-drug resistant *A. baumannii* after phagocytosis by macrophages, and further studies are required to understand the mechanisms (Sato et al., 2019).

We found that TEM and Phagocytosis pathways had some shared up-regulated proteins such as Ncf1, Ncf2, Ncf4, Cybb. Reactome pathway analysis suggested that these proteins were involved in RHO GTPases Activate NADPH Oxidases pathway RHO GTPases (REAC:R-MMU-5668599). NOX is a multi-component enzyme that catalyzes the generation of O^2–^ from oxygen and NADPH. The four essential proteins of NOX subunit were all found significantly up-regulated in *A. baumannii* infected mice lung compared with the control group which demonstrates its critical role in the innate immune response of mice against *A. baumannii* lung infection. Cytochrom b588 composing of gp^91*PHOX*^ (Cybb) and p^22*PHOX*^ (Cyba) serves as the electron transporter. Cytosolic component p^47*PHOX*^ (Ncf1) serves as an adaptor protein, providing a platform for the assembly of cytochrome b588, mediating the interaction both with b588 and p^67*PHOX*^. Protein p^67*PHOX*^ (Ncf2) is another cytosolic component containing two SH3 domains and a NADPH binding domain, which is thought to regulate the transfer electrons from NADPH to FAD. Small GTPase Rac2 also participates in electron transfer (Hordijk, 2006). The fourth component p^40*PHOX*^(Ncf4) contains a PX domain that may bind to special phosphoinositides, thus mediating the assembly of NOX in plasma and cell membrane (Babior et al., 2002). Qiu et al. found that gp^91*phox*–/–^ mice had higher susceptibility to intranasal *A. baumannii* infection than wild-type C57BL/6 mice, with significantly greater bacterial counts in lungs and spleen. It is showed that NOX appears to play a crucial role in host defense against *A. baumannii* (Qiu et al., 2009). Our results similarly indicate that in the host response against *A. baumannii*, NOX is the primary part activated in TEM and Phagocytosis pathways and the NOX-dependent killing mechanism plays an important role.

To observe the bacteria clearance process by host immunity, we used immunocompetent mice in our study. But the immune response is probably different when the host is in a pathogenesis status, a hypervirulent *A. baumannii* strain or immunocompromised mouse could be used to obtain an infection model in a pathogenesis condition, which is closer to a human infection situation in hospitals. Comparing the host response against *A. baumannii* infection under normal and pathogenic status helps to reveal the key factors of the immune system or pathogenesis mechanisms.

In this study, multiple testing was not performed because it was found too conservative for our data resulting in too few candidate proteins. This probably because of the ratio compression of isobaric quantification (Karp et al., 2010). The moderate immune response caused by ATCC19606 and the sampling time could also be the reasons. To validate the up-regulation of candidate proteins Mmp9 and S100a8/a9, we performed Elisa experiments which showed consistent results with the proteomics data. In our future study, high virulence or high bacterial inoculum will be used to observe a more drastic immune response, and the kinetic process of immune response will be studied to determine the effect time of candidate proteins.

In conclusion, in the present study, we used a TMT-labeling quantitative proteomic approach to investigate lung response of mice during bacteria clearance against *A. baumannii.* It is clear that the expression of numerous lung tissue proteins of mice was altered, especially proteins associated with receptor recognition, NADPH oxidase function and antimicrobial peptides, as well as some innate immune pathways including leukocyte transendothelial migration and phagosome. Our results provide a basis for the further study of host-organism interaction and hopefully support the development of treatment strategies of *A. baumannii* human lung infection.

## Data Availability Statement

The mass spectrometry proteomics data have been deposited to the ProteomeXchange with the data set identifier PXD060240.

## Ethics Statement

The animal study was reviewed and approved by the Experimental Animal Ethics Committee of Pharmacy, Fudan University (2019-03-HSYY-ZJ-01).

## Author Contributions

XLi and XLiu designed and performed the experiments, collected, and analyzed the data. XLi wrote the first draft of the manuscript. PH analyzed the data and revised the manuscript. YH revised the manuscript. JZ designed the experiments and revised the manuscript. All authors read and approved the final version of the manuscript.

## Conflict of Interest

The authors declare that the research was conducted in the absence of any commercial or financial relationships that could be construed as a potential conflict of interest.

## Footnotes

1 http://www.chinets.com

2 www.string-db.org

3 https://biit.cs.ut.ee/gprofiler/gost

## Supplementary Material

Supplementary Table 1 List of differentially expressed proteins identified between CON and INF sample group of mice lung.

Supplementary Table 2 Details of the GO, KEGG, and REACTOME pathways obtained from gProfiler.
